# Supplementary material for: Pilot study to explore the use of mobile spaced learning as a digital learning platform when teaching symptom management to undergraduate nursing students: SPLENdidS study
Source: PLoS One. 2022 Jun 8;17(6):e0269633. doi: 10.1371/journal.pone.0269633 (PMC9176778; doi:10.1371/journal.pone.0269633)
Supplement: S1 File — (PDF) [file pone.0269633.s001.pdf]

Questionnaire (Post Intervention) via Survey Monkey

We would like to invite you to take part in evaluating your involvement in online space learning through the digital learning platform when symptom management was being taught as part of the SPLENdidS study. Before you decide whether to take part, you need to understand why we are doing this evaluation and what it will require you to do.

The purpose of this study is to evaluate the use of online space learning activities through the digital learning platform within the undergraduate nursing curriculum. We are particularly interested in understanding your opinion on the usability and acceptability of these learning activities. Information received will contribute to developing these resources which will support students learning during these types of activity.

You are being asked to complete this short confidential online questionnaire. The questionnaire should take approximately 5 to 10 minutes to complete.

If you would like further information, please contact Dr Clare McVeigh by email at clare.mcveigh@qub.ac.uk.

Please indicate your level of agreement with the following usability questions.

1. Technical difficulties significantly reduced my ability to participate.

Strongly Agree Agree Disagree Strongly Disagree

2. This was a good medium for educational activity.

Strongly Agree Agree Disagree Strongly Disagree

3. This tool allowed me to access educational content easily.

Strongly Agree Agree Disagree Strongly Disagree

4. The educational activities were engaging.

Strongly Agree Agree Disagree Strongly Disagree

5. The educational activities were interactive.

Strongly Agree Agree Disagree Strongly Disagree

6. Sufficient time was available for me to answer the questions.

Strongly Agree Agree Disagree Strongly Disagree

7. There were too many questions for each case scenario

Strongly Agree Agree Disagree Strongly Disagree

8. The case scenarios were easy to understand

Strongly Agree Agree Disagree Strongly Disagree

9. The questions were easy to understand

Strongly Agree Agree Disagree Strongly Disagree

10. I enjoyed using the learning platform

Strongly Agree Agree Disagree Strongly Disagree

Did The Learning Tool...

1. Avoid the use of unnecessary jargon.

Strongly Agree Agree Disagree Strongly Disagree

2. Use simple language and short sentences.

Strongly Agree Agree Disagree Strongly Disagree

3. Contain instructions that were clear and unambiguous.

Strongly Agree Agree Disagree Strongly Disagree

4. Provide information that developed my learning on symptom management.

Strongly Agree Agree Disagree Strongly Disagree

5. Contain documents and pages that follow simple and consistent formats.

Strongly Agree Agree Disagree Strongly Disagree

6. Contain no blinking, flashing, or sparkling animated images.

Strongly Agree Agree Disagree Strongly Disagree

I feel that the learning experience…

1. Offered an opportunity to personally learn through participating

Strongly Agree Agree Disagree Strongly Disagree

2. Was enjoyable

Strongly Agree Agree Disagree Strongly Disagree

3. Has improved my skills as a nursing student

Strongly Agree Agree Disagree Strongly Disagree

4. Helps translate knowledge from teaching into practice more than other teaching sessions you have been involved in

Strongly Agree Agree Disagree Strongly Disagree

5. Would be something I would recommend to other students undertaking the BSc Hons Nursing Programme.

Strongly Agree Agree Disagree Strongly Disagree

Please indicate your level of agreement with the following statements

1. I would like to continue to participate in digital learning platforms if the opportunity arose in the future.

Strongly Agree Agree Disagree Strongly Disagree

2. The learning activity was a useful resource for developing my knowledge of symptom management.

What aspect of the experience did you enjoy the most?

What aspect of the experience did you enjoy the least?

Please provide other comments or clarification on your answers above
